# Improving Antibiotic Use for Ventilator-Associated Pneumonia Through Diagnostic Stewardship: A Proof-of-Concept Mixed Methods Study

**DOI:** 10.1093/ofid/ofae500

**Published:** 2024-09-04

**Authors:** Ravi K Tripathi, Blaine Kenaa, Kimberly C Claeys, J Kristie Johnson, Meghana Patel, Jayne Atkinson, Mary E Maldarelli, Michelle Newman, Surbhi Leekha

**Affiliations:** Division of Infectious Diseases, Department of Medicine, School of Medicine, University of Maryland, Baltimore, Maryland, USA; Division of Pulmonary and Critical Care, Department of Medicine, School of Medicine, University of Maryland, Baltimore, Maryland, USA; Department of Practice Science and Health Outcomes Research, School of Pharmacy, University of Maryland, Baltimore, Maryland, USA; Department of Pathology, School of Medicine, University of Maryland, Baltimore, Maryland, USA; Department of Medicine, School of Medicine, University of Colorado, Aurora, Colorado, USA; School of Pharmacy, University of Maryland, Baltimore, Maryland, USA; Division of Pulmonary and Critical Care, Department of Medicine, School of Medicine, University of Maryland, Baltimore, Maryland, USA; Department of Epidemiology and Public Health, School of Medicine, University of Maryland, Baltimore, Maryland, USA; Department of Epidemiology and Public Health, School of Medicine, University of Maryland, Baltimore, Maryland, USA

**Keywords:** diagnostic stewardship, modified laboratory reporting, nudging, VAP overdiagnosis

## Abstract

**Background:**

Overtreatment of ventilator-associated pneumonia (VAP) in the intensive care unit is driven by positive respiratory tract cultures in the absence of a clinical picture of pneumonia. We evaluated the potential for diagnostic stewardship at the respiratory culture reporting step.

**Methods:**

In this mixed methods study, we conducted a baseline evaluation of lower respiratory tract (LRT) culture appropriateness and antibiotic prescribing, followed by a nonrandomized intervention in 2 adult intensive care units. The intervention was a comment in the report to indicate potential colonization instead of organism identification when LRT cultures were inappropriate—that is, not meeting criteria for pneumonia as adjudicated by a physician using a standard algorithm.

**Results:**

At baseline, among 66 inappropriate LRT cultures, antibiotic treatment for VAP was more frequent with identification of potential pathogens in the index culture when compared with no growth/normal flora (16/35 [46%] vs 7/31 [23%], *P* = .049). In the intervention period, 28 inappropriate cultures with growth of potential pathogens underwent report modification. The proportion of episodes for which antibiotic therapy for VAP was completed was significantly lower in the intervention group vs the baseline group (5/28 [18%] vs 16/35 [46%], *P* = .02).

**Conclusions:**

Diagnostic stewardship for VAP could be facilitated by modification of LRT culture reporting guided by clinical features of pneumonia.

## Teaser

Identification of organisms growing in respiratory cultures (ie, “positive culture”) drives antibiotic treatment even in the absence of clinical findings of ventilator-associated pneumonia. Modifying laboratory reporting based on clinical findings may reduce ventilator-associated pneumonia overdiagnosis and unnecessary antibiotics.

Overdiagnosis of ventilator-associated pneumonia (VAP) is common and may be due, in part, to excessive culturing of the lower respiratory tract (LRT) in patients who are ventilated [1–3]. Nonspecific clinical and radiographic features in a disease that has high mortality, combined with sepsis vigilance, contribute to excessive use of LRT culturing and VAP overdiagnosis in 40% to 70% of patients [2, 4–6]. While antimicrobial stewardship is instrumental in guiding providers in appropriate antibiotic selection and limiting unnecessary therapy, it relies heavily on culture results and does not address positive respiratory cultures sent in the setting of a low pretest probability for true infection. Diagnostic stewardship intervenes upstream of antimicrobial stewardship by preventing a positive culture report and bacterial identification in a patient who is colonized without true clinical signs of infection [7]. Modified laboratory reporting has been particularly successful in decreasing the inappropriate treatment of asymptomatic bacteriuria and in de-escalation of antipseudomonal and anti–methicillin-resistant *Staphylococcus aureus* antibiotics in respiratory tract infections [8–10].

In this study, we sought to strengthen the rationale for intervening at the respiratory culture reporting step. Specifically, we wanted to evaluate the impact of a pilot diagnostic stewardship intervention of modified laboratory reporting of respiratory cultures, conditioned on the clinical likelihood of pneumonia, on antibiotic use in patients undergoing ventilation in the intensive care unit (ICU). This study had the following 2 aims: (1) to conduct a baseline evaluation of the appropriateness of LRT culture ordering and antibiotic use in relation to final culture result reports and (2) to evaluate a nonrandomized diagnostic stewardship intervention of culture reporting on patient outcomes and antibiotic use.

## METHODS

### Study Design and Setting

We conducted a mixed methods proof-of-concept study piloting a diagnostic stewardship intervention leveraging respiratory culture order review and conditional modification of respiratory culture reporting at an 800-bed academic medical center in Baltimore, Maryland. We conducted the baseline evaluation and nonrandomized intervention among adult patients in 2 ICUs: the neuro-care ICU (20 beds) and cardiac surgery ICU (22 beds). Patients were included if they had LRT cultures obtained from endotracheal aspirate, bronchoscopy with bronchoalveolar lavage, bronchial washing, or mini bronchoalveolar lavage, following ≥48 hours of mechanical ventilation. Heart and lung transplant recipients and patients undergoing extracorporeal membrane oxygenation were excluded. Multiple respiratory cultures were eligible for study inclusion during the same patient admission. VAP prevention measures, including oral care, head-of-bed elevation, and spontaneous breathing trials, were in place in both ICUs per institutional policy. The study was approved by the University of Maryland Baltimore Institutional Review Board as minimal risk (HP-00082703).

### Baseline Study Period

**Figure 1.**
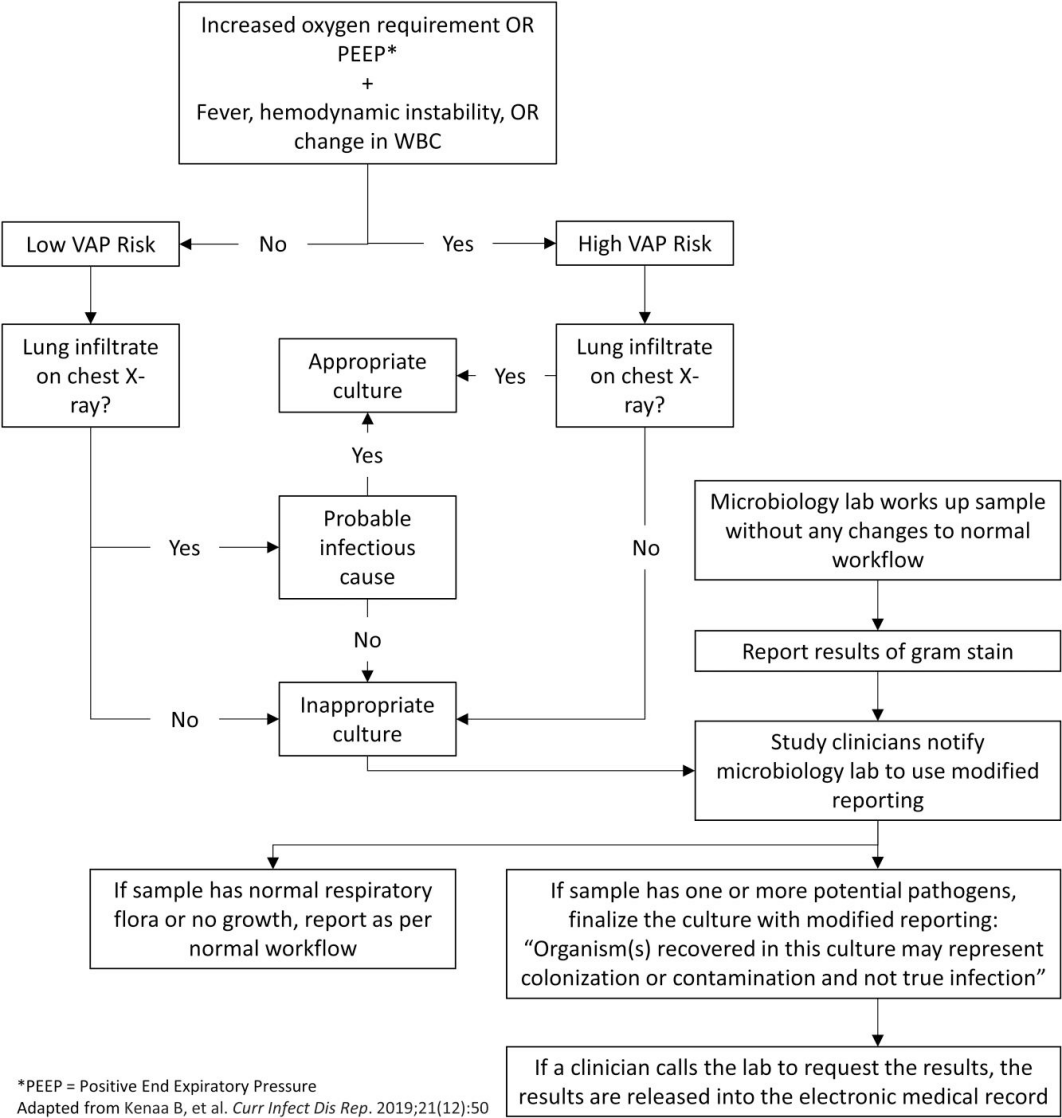
Study algorithm for evaluation of appropriateness of lower respiratory tract cultures. PEEP, positive end-expiratory pressure; VAP, ventilator-associated pneumonia; WBC, white blood cell.

Preintervention (baseline) evaluation of the appropriateness of LRT culture ordering was conducted between 1 March and 30 June 2021 in the 2 study ICUs, when COVID-19 activity was at its lowest. Cultures were retrospectively evaluated for the appropriateness of ordering by 1 of 2 physicians, one trained in pulmonary and critical care medicine and the other in infectious diseases, through electronic medical record (EMR) review via a previously published diagnostic algorithm [11]. This algorithm uses the following clinical and laboratory attributes to determine the probability of pneumonia: worsening gas exchange (increase in fraction of inspired oxygen by 0.20 or increase in positive end-expiratory pressure by 3 cm H_2_0, sustained over 6 hours), fever (>38 °C), leukocytosis or leukopenia (white blood cell count >12 or <4 × 10^9^/L), hemodynamic instability (defined as the initiation of vasopressor agents or an acute escalation in dose), and infiltrates or opacities on chest imaging (Figure 1). Radiographic findings were interpreted by formal radiologist readings, followed by the treating teams' impression in their progress notes with consideration for new and persistent infiltrates. Prior to the start of the study, a few cases were reviewed by the study team physicians to refine the application of the algorithm and ensure standardization of the review process. Antibiotic therapy associated with each index LRT culture was classified into 1 of 6 mutually exclusive categories:

A: Not prescribed empiric antibiotics, new antibiotic started per index respiratory culture result
B: Not prescribed empiric antibiotics, no new antibiotics initiated within 3 days of culture result
C: Prescribed empiric antibiotics, changed per index respiratory culture, completed course for VAP
D: Prescribed empiric antibiotics, continued without change following index respiratory culture, completed course for VAP
E: Prescribed empiric antibiotics, antibiotics discontinued per culture results
F: Prescribed antibiotics for another condition or diagnosis, but this therapy was not directed at the results of the index respiratory culture

Antibiotic use falling into categories A, C, and D constituted antibiotic treatment for VAP. During the baseline period, standard hospital laboratory practices remained unchanged. All respiratory cultures were first assessed for quality by the presence of squamous epithelial cells and polymorphonuclear white cells present on Gram stain. Potential pathogens were identified by the specific organisms, number of unique organisms, and quantification relative to other organisms.

### Diagnostic Stewardship Intervention: Implementation of Modified Reporting

The intervention period was 1 May through 15 November 2022, after COVID-19 activity had decreased substantially following the first Omicron wave from December 2021 to March 2022. Therefore, baseline and intervention periods were asynchronous to avoid surges in COVID-19. During the intervention period, LRT culture orders were prospectively reviewed within 24 hours of order placement Monday through Friday by 1 of the 2 trained physician reviewers. In addition, any equivocal cases were discussed between the physician reviewers ad hoc. The microbiology laboratory respiratory bench was notified by phone of any cultures where appropriateness criteria were not met; notification occurred prior to the first reporting of culture results in the EMR. The technician followed usual workflow for respiratory culture workup, except that in the presence of dominant growth of 1 or more organisms that would normally be reported, the following statement was reported in lieu of organism identification in the EMR: “Organism(s) recovered in this culture may represent colonization or contamination and not true infection.” Bedside clinicians were blinded to the intervention; however, they could request the full report of organism identification and antibiotic susceptibility information by calling the microbiology laboratory, and the final decision to treat for VAP remained at the discretion of the primary team. There was no direct interaction between the study team and treating providers.

### Outcome Definitions and Measures

In the baseline group only, we calculated the overall proportion of inappropriate LRT cultures and compared the frequency of each clinical attribute between inappropriate and appropriate cultures. Among the subset of cultures considered inappropriate, we compared the proportion treated for VAP between the following 2 groups: those where final culture results were reported as “no growth” or “normal respiratory flora” and those where at least 1 potential pathogen was identified. In the pre- and postintervention analysis, the primary outcome evaluating the impact of the diagnostic stewardship intervention was the proportion of cultures for which antibiotic treatment for VAP was prescribed in the baseline vs intervention periods. Cultures were eligible for analysis if determined to be inappropriate and reported with growth of 1 or more potentially pathogenic organisms (Supplementary Figure 1 shows study enrollment overview).

We hypothesized a reduction in the proportion of LRT cultures for which antibiotic therapy for VAP was prescribed. A secondary antibiotic outcome was patient-level length of antibiotic therapy related to the index LRT culture. The length of antibiotic treatment was defined as uninterrupted calendar days of therapy, irrespective of the number of different antibiotics, from first start of empiric therapy to stop, allowing for a maximum of 28 days or hospital discharge, whichever occurred first, and was determined from medication administration data in the EMR.

To evaluate potential adverse outcomes from missed VAP diagnoses, we measured the incidence of septic shock (as defined by the treating team) and death, both assessed between days 0 and 4 and between days 5 and 14 from the index LRT culture. All episodes of septic shock in the intervention group were assessed for potential relatedness to the intervention. Additional relevant clinical outcomes included duration of mechanical ventilation, duration of ICU stay, and in-hospital 28-day mortality. As a balancing measure, to assess if the modified comment would lead to an increase in repeat LRT culture, we evaluated the frequency of repeat cultures within 3 days of the index culture. We hypothesized no difference in the incidence of these events between the baseline and intervention groups. This pilot study was intended to inform the feasibility and sample size for a larger cluster-randomized study; therefore, a sample size calculation was not done. All data were collected by a REDCap data capture tool [12].

### Statistical Analyses

In the baseline period, the proportion of inappropriate cultures treated as VAP were compared between those with growth of a potential pathogen and those with no growth/normal flora by chi-square or Fisher exact test. Baseline and intervention groups were compared by chi-square test for nominal categorical variables and Student *t* test or Wilcoxon rank sum test for continuous data, as appropriate. Logistic regression odds ratios were calculated for all clinical outcomes of interest, except that median antibiotic length of therapy between baseline and intervention groups was compared with a Wilcoxon rank sum test. We used multivariable logistic regression analysis to calculate odds of antibiotic treatment between the baseline and intervention groups, adjusting for covariates that were significantly different between them or a priori considered potentially able to affect the outcome.

## RESULTS

### Baseline LRT Culturing Characteristics and Antibiotic Therapy

**Figure 2.**
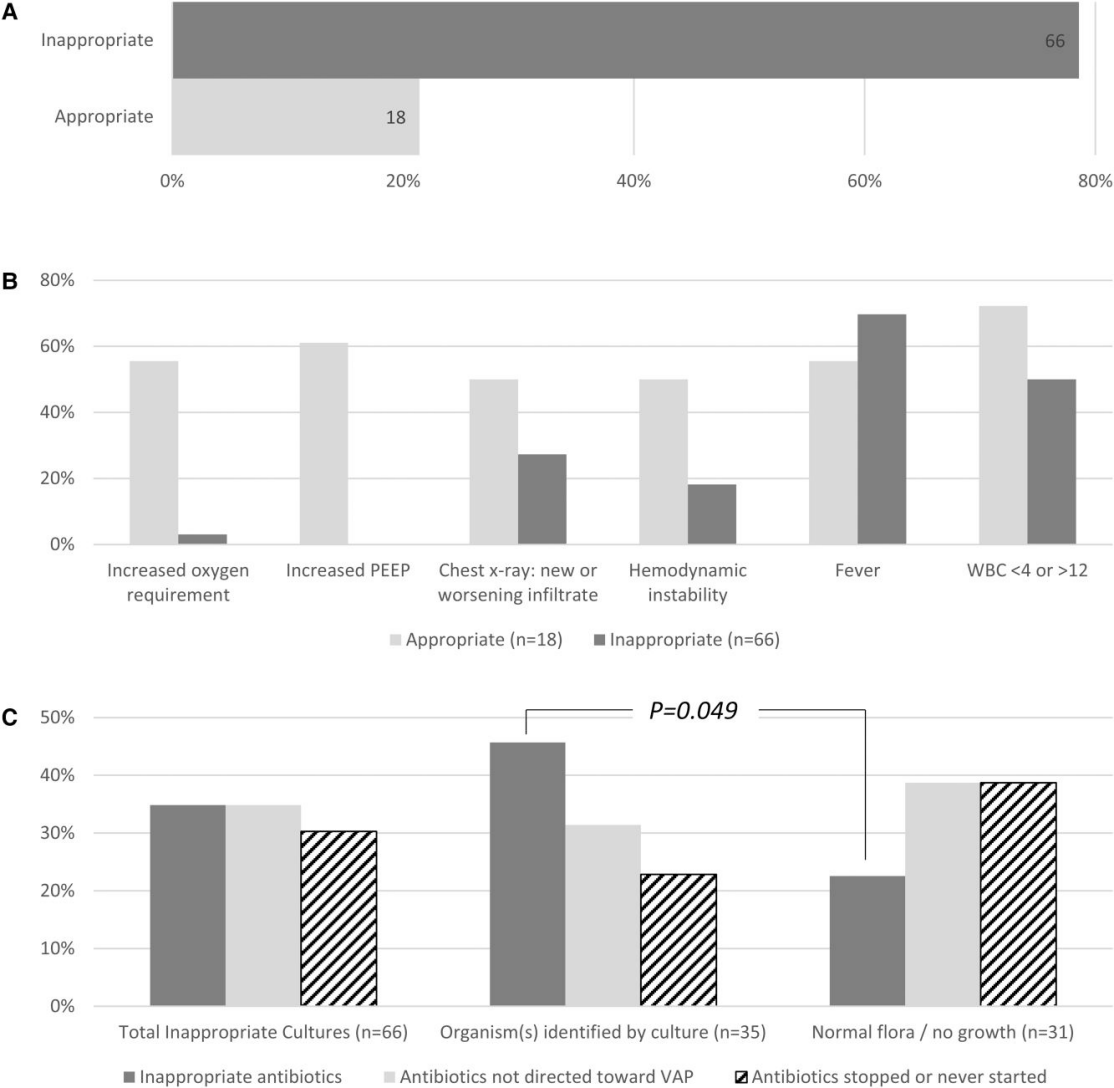
*A*, Proportion of lower respiratory tract cultures considered appropriate or inappropriate as determined by a physician using a study algorithm. *B*, Comparison of clinical attributes of appropriate and inappropriate lower respiratory tract cultures. *C*, Antibiotic use characteristics among inappropriate lower respiratory tract cultures overall and by cultures with potentially pathogenic organisms identified in the culture report vs cultures with normal flora or no growth. PEEP, positive end-expiratory pressure; VAP, ventilator-associated pneumonia; WBC, white blood cell.

In the baseline period, 84 LRT cultures were evaluated, of which 66 (79%) were considered inappropriate (Figure 2*A*). The algorithm was most likely to distinguish patients with appropriate cultures from those with inappropriate cultures based on worsening oxygenation or increased positive end-expiratory pressure, followed by hemodynamic instability and radiographic changes, with smaller differences between groups for fever and white blood cell count (Figure 2*B*). Of the 66 LRT cultures considered inappropriate, there were 38 endotracheal aspirates, 18 mini bronchoalveolar lavages, 8 bronchoalveolar lavages, and 2 bronchial washings. Overall, 23 of 66 (35%) completed antibiotic treatment for VAP. Of the 66 inappropriate cultures, 35 (53%) had 1 or more potential pathogens identified. Among those with 1 or more potentially pathogenic organisms identified, 16 of 35 (46%) completed antibiotic treatment for VAP as compared with 7 of 31 (23%) with no growth/normal flora (*P* = .049; Figure 2*C*).

### Comparison of Baseline and Intervention Groups

Among 114 LRT cultures (from 97 patients) considered inappropriate, there were 66 cultures in 56 patients in the baseline period and 48 cultures in 41 patients in the intervention period. The proportion of cultures reported with growth of 1 or more potentially pathogenic organisms was similar between the baseline group (35/66, 53%) and intervention group (28/48, 58%). Thus, 35 LRT cultures (cultures eligible for intervention) among 28 patients in the baseline group were compared with 28 index cultures (cultures intervened upon) among 22 patients in the intervention group (Supplementary Figure 1). Patients in these 2 groups were similar with respect to demographic and clinical characteristics, except that a higher proportion of patients in the intervention group had undergone surgery in the previous 30 days (Table 1). The median time from hospital admission to index LRT culture was longer (11.5 days [IQR, 6–16.5] vs 5 days [IQR, 3–12]; *P* = .083), and the presence of infectious disease consultation at the time of the index culture was more frequent (59% vs 41%, *P* = .057) in the intervention group as compared with the baseline group.

**Table 1.** Demographic and Clinical Characteristics of Patients With Inappropriate Lower Respiratory Tract Cultures (N = 97)

	Baseline Period			Intervention Period			
	Total (n = 56)	Normal/No Growth: Not Eligible for Intervention (n = 28)	Potential Pathogen Reported: Eligible for Intervention (n = 28)	Total (n = 41)	No Intervention: Normal Flora/No Growth (n = 19)	Intervention: Potential Pathogens Recovered but Not Reported (n = 22)	*P* Value^a^
Demographics							
Age, y, mean (SD)	61 (16)	62 (17)	60 (15)	64 (14)	66 (14)	62 (14)	.41
Female sex	21 (37.5)	8 (28.6)	13 (46.4)	16 (39)	7 (36.8)	9 (40.9)	.69
CHF	16 (28.6)	7 (25)	9 (32.1)	14 (34.1)	9 (47.4)	5 (22.7)	.6
COPD	9 (16.1)	2 (7.1)	7 (25)	6 (14.6)	2 (10.5)	4 (18.2)	.73
Organ transplant recipient	1 (1.8)	1 (3.6)	0 (0)	0 (0)	0 (0)	0 (0)	…
Chronic liver disease	3 (5.4)	1 (3.6)	2 (7.1)	1 (2.4)	1 (5.3)	0 (0)	.49
Diabetes mellitus	27 (48.2)	13 (46.4)	14 (50)	13 (31.7)	7 (36.8)	6 (27.3)	.1
Active malignancy	1 (1.8)	0 (0)	1 (3.6)	1 (2.4)	0 (0)	1 (4.5)	>.99
HIV	…	…	…	…	…	…	…
Smoking/tobacco use	14 (25)	5 (17.9)	9 (32.1)	13 (31.7)	4 (21.1)	9 (40.9)	.56
Obesity	30 (53.6)	16 (57.1)	14 (50)	15 (36.6)	7 (36.8)	8 (36.4)	.34
Other chronic lung disease	7 (12.5)	2 (7.1)	5 (17.9)	5 (12.2)	2 (10.5)	3 (13.6)	.98
Hospital episode							
ICU location							>.99
CSICU	22 (39.3)	11 (39.3)	11 (39.3)	22 (53.7)	13 (68.4)	9 (40.9)	
NCCU	34 (60.7)	17 (60.7)	17 (60.7)	19 (46.3)	6 (31.6)	13 (59.1)	
COVID-19 status							.24
Negative this admission	54 (96.4)	28 (100)	26 (92.9)	37 (90.2)	16 (84.2)	21 (95.5)	
Active infection	2 (3.6)	0 (0)	2 (7.1)	1 (2.4)	1 (5.3)	0 (0)	
Recovered	0 (0)	0 (0)	0 (0)	3 (7.3)	2 (10.5)	1 (4.5)	
ARDS	10 (17.9)	5 (17.9)	5 (17.9)	4 (9.8)	3 (15.8)	1 (4.5)	.21
CPR within 72 h	3 (5.4)	1 (3.6)	2 (7.1)	1 (2.4)	1 (5.3)	0 (0)	.49
Immunosuppressive therapy							.49
Short term, <30 d	2 (3.6)	1 (3.6)	1 (3.6)	0 (0)	0 (0)	0(0)	
Long term, >30 d	2 (3.6)	1 (3.6)	1 (3.6)	2 (4.9)	0 (0)	2 (9.1)	
Neutropenia	…	…	…	…	…	…	…
Within 30 d prior							
Shock	20 (35.7)	8 (28.6)	12 (42.9)	24 (58.5)	12 (63.2)	12 (54.5)	.41
Trauma	2 (3.6)	2 (7.1)	0 (0)	2 (4.9)	1 (5.3)	1 (4.5)	.44
Surgery or procedure performed	29 (51.8)	14 (50)	15 (53.6)	34 (82.9)	17 (89.5)	17 (77.3)	.08

Data are presented as No. (%) unless noted otherwise. In total, 97 unique patients contributed 114 lower respiratory tract cultures; for patient-level analyses, only data from the time of first culture during the study period were included.

Abbreviations: ARDS, acute respiratory distress syndrome; CHF, congestive heart failure; COPD, chronic obstructive pulmonary disease; CPR, cardiopulmonary resuscitation; CSICU, cardiac surgery intensive care unit; ICU, intensive care unit; NCCU, neurocritical care unit.

^a^ *P* value for difference between baseline and intervention potential pathogens; chi-square or Fisher exact test.

Among those eligible for the intervention, the proportion of index LRT cultures for which antibiotic therapy for VAP was completed was significantly lower in the intervention group (5/28, 18%) vs the baseline group (16/35, 46%; *P* = .02; Table 2). Controlling for infectious disease consultation, those in baseline group had 5-fold increased odds of completing VAP treatment as compared with the intervention group (adjusted odds ratio, 4.98; 95% CI, 1.40–17.66). The median antibiotic length of therapy was nonsignificantly lower in the intervention when compared with baseline (3 days [IQR, 0–10.8] vs 5.5 days [IQR, 1–8.3]; *P* = .31).

**Table 2.** Baseline and Intervention Antibiotic, Clinical, and Safety Outcomes Related to Inappropriate Lower Respiratory Tract Cultures With Potentially Pathogenic Organisms (n = 63)

	Baseline: Eligible for Intervention (n = 35)	Intervention: Modified Report (n = 28)	Odds Ratio (95% CI) or *P* Value
Clinical diagnosis and completed treatment of VAP^a^	16 (45.7)	5 (17.9)	3.9 (1.2–12.5)
Breakdown of antibiotic treatment of VAP			
Group A^b^	5 (14.3)	0 (0)	10.3 (.5–194.4)
Group C^c^	7 (20.0)	2 (7.1)	3.3 (.6–17.1)
Group D^d^	4 (11.4)	3 (10.7)	1.1 (.2–5.3)
Antibiotic therapy for other indication: group F	11 (31.4)	10 (35.7)	0.8 (.3–2.4)
Bacteremia			
Days 1–4	2 (5.7)	4 (14.3)	0.4 (.06–2.1)
Days 5–14	5 (14.3)	3 (10.7)	1.4 (.3–6.4)
Septic shock			
Days 1–4	6 (17.1)	2 (7.7)	2.7 (.5–14.5)
Days 5–14	2 (5.7)	5 (17.9)	…
Weaned from ventilation: days 5–14	8 (22.9)	3 (10.7)	2.46 (.6–10.4)
Discharge disposition: day 28			
Home	2 (5.7)	2 (7.1)	0.8 (.1–5.9)
Hospice	2 (5.7)	2 (7.1)	0.8 (.1–5.9)
Rehabilitation	5 (14.3)	6 (21.4)	0.6 (.2–2.2)
Skilled nursing facility	3 (8.6)	3 (10.7)	0.7 (.1–4.2)
Transfer outside hospital	1 (2.9)	4 (14.3)	2 (.02–1.7)
Remained hospitalized	13 (37.1)	6 (21.4)	2.4 (.8–7.3)
Inpatient mortality by day 28	9 (25.7)	5 (17.9)	1.6 (.5–5.4)
Median (IQR)			
Postculture days of mechanical ventilation^e^	8 (4–11)	8 (4–10.5)	>.99
Overall LOS, d	35 (24.25–75)	54.5 (27–92.75)	.307
Postculture LOS, d	25 (12–47)	34 (10–50)	.212

Data are presented as No. (%) unless noted otherwise. In total, there were 63 lower respiratory tract cultures among 50 unique patients: 28 patients in baseline and 22 in intervention.

Abbreviations: LOS, length of stay; VAP, ventilator-associated pneumonia.

^a^Clinical diagnosis of VAP represents whether the treating team assigned a diagnosis in the chart in the first 4 days after lower respiratory tract culture was obtained.

^b^Group A: Not prescribed empiric antibiotics, new antibiotic started per index respiratory culture result.

^c^Group C: Prescribed empiric antibiotics, changed per index respiratory culture, completed course for VAP.

^d^Group D: Prescribed empiric antibiotics, continued without change following index respiratory culture, completed course for VAP.

^e^Within 14 days of index culture.

In the intervention group, 3 of 28 cultures had the organism identification released upon request, and an additional 5 had the organism identification inadvertently released in addition to the modified report comment. Of these, 2 of 8 (25%) received antibiotic therapy for VAP. In a sensitivity analysis, after these 8 cultures were excluded from the intervention group (n = 20), the difference between the baseline and intervention groups in proportion treated for VAP remained relatively unchanged (46% [16/35] vs 15% [3/20], *P* = .021).

When relevant clinical outcomes were evaluated, there was no significant difference in the incidence of bacteremia or septic shock within 4 days or during days 5 to 14 after the index LRT culture between baseline and intervention (Table 2). None of the episodes of septic shock in the intervention group were related to a missed or delayed diagnosis of VAP. Similarly, there were no significant differences in mechanical ventilation, ICU discharge, or 28-day mortality. The overall hospital length of stay was slightly longer in intervention as compared with baseline, with a longer duration prior to and after the index LRT culture, but this was not statistically significant. We found no differences in the frequency of repeat culturing within 3 days of index culture between the intervention and baseline periods (6 [21%] vs 8 [23%], *P* = .892).

## DISCUSSION

In this single-center 2-ICU study, we found that identification of organisms growing in LRT cultures (ie, “positive culture”) drives antibiotic treatment for VAP even in the absence of clinical findings of pneumonia. In a nonrandomized intervention, in instances of potentially inappropriate culturing, modification of culture reporting to indicate the likelihood of colonization in lieu of organism identification was associated with a significant reduction in antibiotic treatment for VAP, without an increase in adverse events.

The need for diagnostic stewardship for infectious diseases is becoming increasingly recognized [13, 14]. In our baseline period, we found that LRT cultures were frequently ordered in the absence of clinical suspicion for pneumonia. This is similar to previous findings of “pan-culturing” in the ICU being frequently triggered by fever, leukocytosis, and concern for sepsis, even in the absence of site-specific findings [2, 15, 16]. A previous study compared propensity score–stratified cohorts of patients who were mechanically ventilated and had a new abnormal temperature or white blood cell count but without radiographic changes of pneumonia, an increase in ventilator settings, or purulent secretions. It found that patients with respiratory cultures performed within 36 hours of those changes were more likely to receive antibiotics within 1 week when compared with those not undergoing respiratory culturing [16].

As with asymptomatic bacteriuria, asymptomatic respiratory tract colonization without pneumonia or “asymptomatic bacterisputia” is common, with gram-negative bacterial colonization in approximately 50% of patients by day 5 of mechanical ventilation [3, 17]. In our baseline review of respiratory cultures, 46% of patients with an inappropriately ordered culture and bacterial pathogen identified on culture report were treated for possible VAP, as compared with 23% of patients with inappropriately ordered culture but no growth/normal flora, underscoring the “positive” culture result as a stimulus to treat [18]. This is consistent with our prior qualitative study where health care providers demonstrated a lower propensity toward VAP treatment for normal flora as compared with recovery of a potential pathogen [1]. Accordingly, our modified comment was designed to mimic the reporting of normal flora and suggestion of colonization. This is similar to findings in asymptomatic bacteriuria where health care providers often treat positive urine cultures in the absence of symptoms of urinary tract infection and are more likely to treat positive cultures with certain organisms and with higher colony counts [19–22]. Furthermore, an intervention of modified reporting of urine cultures—where providers were asked to call the laboratory to request specifics of positive urine cultures if urinary tract infection was clinically suspected—was associated with a significant reduction in inappropriate antibiotic treatment [8, 23].

The decision to intervene at the reporting step was informed by our earlier qualitative work studying respiratory tract culturing among ICU clinicians, where we found high diagnostic value of fever and hypotension, as well as culturing driven by sepsis vigilance and fear of missing serious diagnoses [1, 4]. Unlike alerts or reminders at the step of culture ordering, which can interrupt clinical workflow, lead to alert fatigue, or be ignored, comments in laboratory results can nudge clinicians toward desired behavior without being disruptive [13, 24, 25]. Although they require clinical review, when compared with prospective antibiotic audit and feedback, these can be potentially timesaving, as there is no direct interaction between the stewardship team and clinician, while preserving perceived prescribing autonomy.

We evaluated the possibility of patient harm from a potentially missed VAP diagnosis by evaluating the incidence of bacteremia and septic shock following the index respiratory culture. We did not find a difference between the intervention and baseline groups or any episode of septic shock in the intervention group related to a missed diagnosis of VAP.

Strengths of this study include a novel intervention supported by theoretical and actual prescribing behavior relative to culture reporting, measurement of its impact on antibiotic use, and evaluation of intervention safety. Limitations include a single-center nonrandomized intervention, as institution-specific biases and protocols may drive culturing and antibiotic treatment. However, similar experiences have been described in studies from other institutions in adult and pediatric patients [26, 27]. Our intervention did not limit respiratory culture ordering or change the reporting of initial Gram stain, which could influence antibiotic prescribing prior to final culture results. To conduct the study during the period of lowest COVID-19 activity in both years, we included different calendar months in the baseline and intervention periods. Although overall antibiotic use may be affected by secular, seasonal trends, our antibiotic use outcomes were assessed in relation to the index respiratory culture order and unlikely to be affected by overall seasonal variation. Finally, although this intervention requires less time than a discussion with the treating provider, it is still resource intensive with respect to expert review for assessment of culturing appropriateness. Future studies should employ a randomized multicenter design and evaluate whether such an intervention is sustainable and, particularly, if clinical reviews could be performed more efficiently by leveraging the EMR for automation.

## CONCLUSION

In this proof-of-concept study, potentially inappropriate LRT cultures, especially when reported as “positive” for pathogenic organisms, are associated with overdiagnosis and overtreatment of VAP in the adult ICU setting. In a nonrandomized intervention conducted in 2 ICUs among patients with low likelihood of VAP, modification of respiratory culture result reporting to indicate potential colonization was associated with a significant reduction in antibiotic therapy for VAP as compared with baseline, without evidence of an increase in adverse events. Modification of respiratory culture reporting conditioned on a patient's clinical presentation may be an effective and safe method of diagnostic and antimicrobial stewardship in the ICU setting and should be studied on a larger scale.

## Supplementary Material

ofae500_Supplementary_Data

## Supplementary Data

Supplementary materials are available at *Open Forum Infectious Diseases* online. Consisting of data provided by the authors to benefit the reader, the posted materials are not copyedited and are the sole responsibility of the authors, so questions or comments should be addressed to the corresponding author.

## Notes

***Acknowledgments*.** We thank Reema Andrade and Wenduo Lai for their contributions to the study.

***Author contributions***. Study conception and design: R. K. T., B. K., K. C. C., J. K. J., M. N., S. L. Data collection: R. K. T., B. K., M. P., J. A., M. E. M. Analysis and interpretation of result: R. K. T., B. K., K. C. C., M. N., S. L. Draft manuscript and preparation: R. K. T., B. K., K. C. C., S. L. Critical revision and final approval: R. K. T., B. K., K. C. C., J. K. J., M. P., M. E. M., J. A., M. N., S. L.

***Availability of data and materials.*** The data sets used and/or analyzed during the current study are available from the corresponding author on reasonable request.

***Patient consent*.** Ethics approval and consent to participate: not applicable. This study was approved as minimal risk by the Institutional Review Board of the University of Maryland.
