# Supplementary figures and images for: Improving Antibiotic Use for Ventilator-Associated Pneumonia Through Diagnostic Stewardship: A Proof-of-Concept Mixed Methods Study
Source: Open Forum Infect Dis. 2024 Sep 4;11(9):ofae500. doi: 10.1093/ofid/ofae500 (PMC11420684; doi:10.1093/ofid/ofae500)

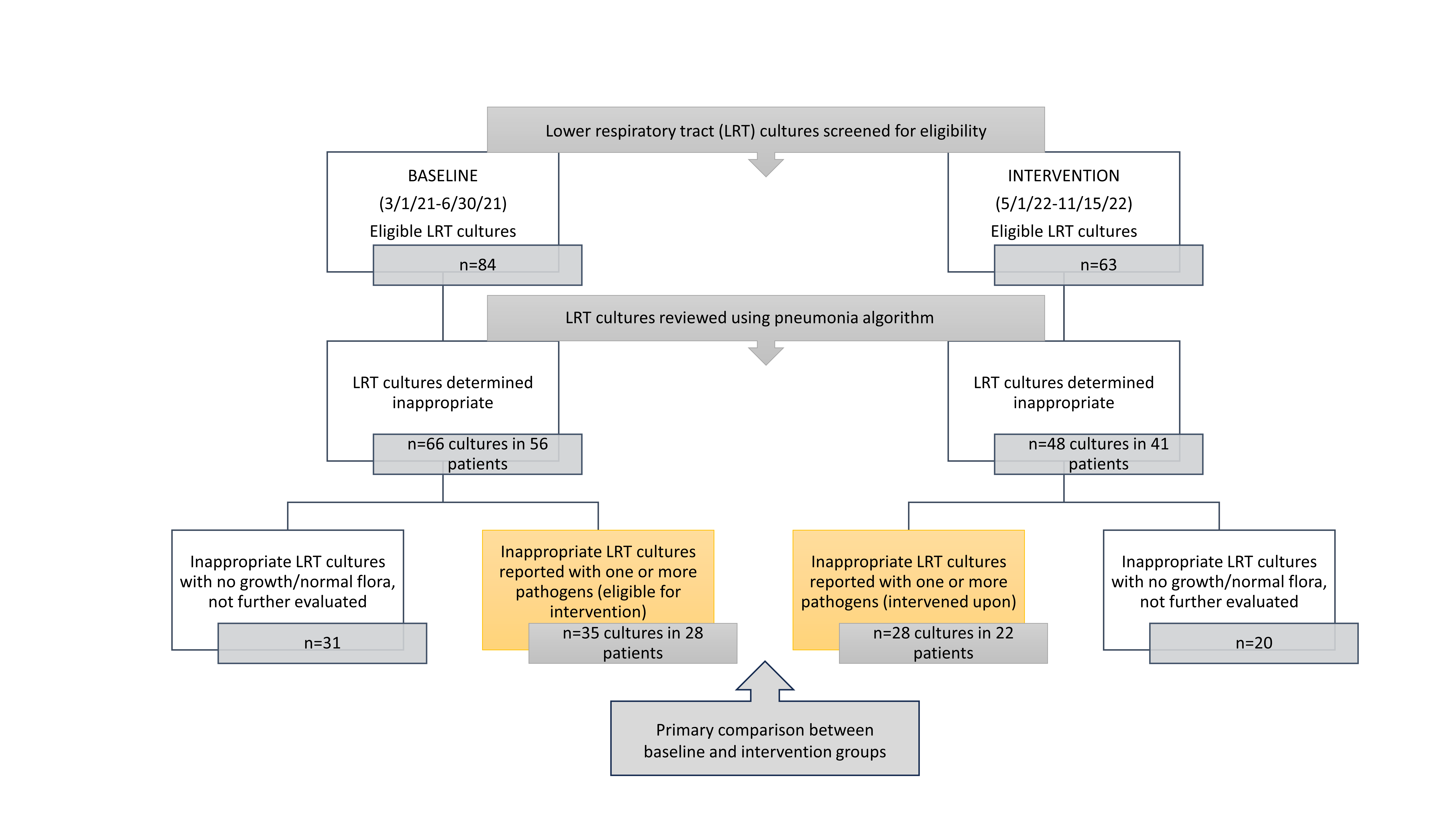

Supplement: ofae500_Supplementary_Data [file ofae500_supplementary_data.zip › Supplemental Figure 1 - Final.tif]
